# Protective effects of rutin against potassium bromate induced nephrotoxicity in rats

**DOI:** 10.1186/1472-6882-12-204

**Published:** 2012-11-01

**Authors:** Rahmat Ali Khan, Muhamad Rashid Khan, Sumaira Sahreen

**Affiliations:** 1 Department of Biotechnology, Faculty of Biological Sciences, University of Science and Technology Bannu, Khyber Pakutunkhwa, Pakistan; 2 Department of Biochemistry, Faculty of Biological Sciences, Quaid-i-Azam University, Islamabad, Pakistan; 3 Botanical Sciences Division, Pakistan Museum of Natural History, Garden Avenue, Shakarparian, Islamabad, Pakistan

**Keywords:** Potassium bromate, Rutin, Histopathology, Renal function test, Antioxidant, DNA fragmentation, Telomerase

## Abstract

**Background:**

Rutin, a polyphenolic flavonoid, was investigated for its protective effects against the KBrO_3_ induced renal injuries in rat.

**Methods:**

Group I was control (untreated), group II was given saline 0.5 ml/kg bw (0.9% NaCl), group III was administered KBrO_3_ (20 mg/kg bw) intragastric twice a week for four weeks. Rutin was administered to group VI (50 mg/kg bw) and Group V (70 mg/kg bw) along with KBrO_3_ (20 mg/kg bw) while group VI was given rutin (70 mg/kg bw) alone twice a week for four weeks. Protective effects of rutin on KBrO_3_-induced nephrotoxicity in rats were determined for biochemical parameter of urine, and serum, various antioxidant enzymes, DNA and histopathological damages in kidneys.

**Results:**

The level of urinary red blood cells, leucocytes count, specific gravity, urea, creatinine and urobilinogen was increased (P<0.01) whereas creatinine clearance was reduced. Serum level of protein, albumin, globulin, nitrite, creatinine and blood urea nitrogen (BUN) was significantly increased (P<0.01) by KBrO_3_. Marked histopathological lesions, elevated DNA fragmentation and AgNORs count in renal tissues was determined. Activity of antioxidant enzymes; catalase, superoxide dismutase, glutathione peroxidase, glutathione-S-transferase, glutathione reductase, and reduced glutathione contents were decreased (P<0.01) while thiobarbituric acid reactive substances were increased (P<0.01) with KBrO_3_ treatment in kidneys. DNA ladder assay was intimately related with the DNA fragmentation assay. Telomerase activity was found positive in the KBrO_3_ treated kidneys. Treatment with rutin effectively ameliorated the alterations in the studied parameters of rat. Rutin administration alone to rats did not exhibit any significant change in any of the parameters studied.

**Conclusion:**

These results suggest that rutin works as an antioxidant *in vivo* by scavenging reactive oxygen species and this serves to prevent oxidative renal damage in rat treated with KBrO_3_.

## Background

Potassium bromate (KBrO_3_) has been used widely for water disinfection, hair-coloring solutions, cosmetics, and in food [1]. Toxicological studies have suggested that KBrO_3_ is: an oxidizing agent; causes hepatotoxicity, neurotoxicity, and thyroid toxicity; and induces the development of mesothelioma tumors in experimental animals as well as renal carcinomas in animals and humans [2,3]. Several studies have investigated the oxidative injuries and probable mechanism of KBrO_3_-induced carcinogenicity in experimental models [4-6]. KBrO_3_ induces mutations, base modification (8-oxodeoxyguanosine), chromosomal aberrations, and alters gene expression, leading to cancer [7-9]. KBrO_3_ increases lipid peroxidation, the creatinine concentration, and enzyme activity in the sera of rats. Cellular defense against oxidative stress is provided by several mechanisms. Antioxidant enzymes as well as non-enzymatic compounds such as reduced glutathione (GSH), ascorbic acid, and α-tocopherol all help to cope with the potential damage caused by oxidative stress [10]. Free radicals produced by KBrO_3_ are associated with the activation of telomerase activity. This activation induces neoplasia, hyperplasia and tumor formation [11,12], increases the number and size of argyrophilic nucleolar organizer regions (AgNORs) and can be utilized as an indicator of genotoxicity to complement other histological procedures [13,14].

**Figure 1:**
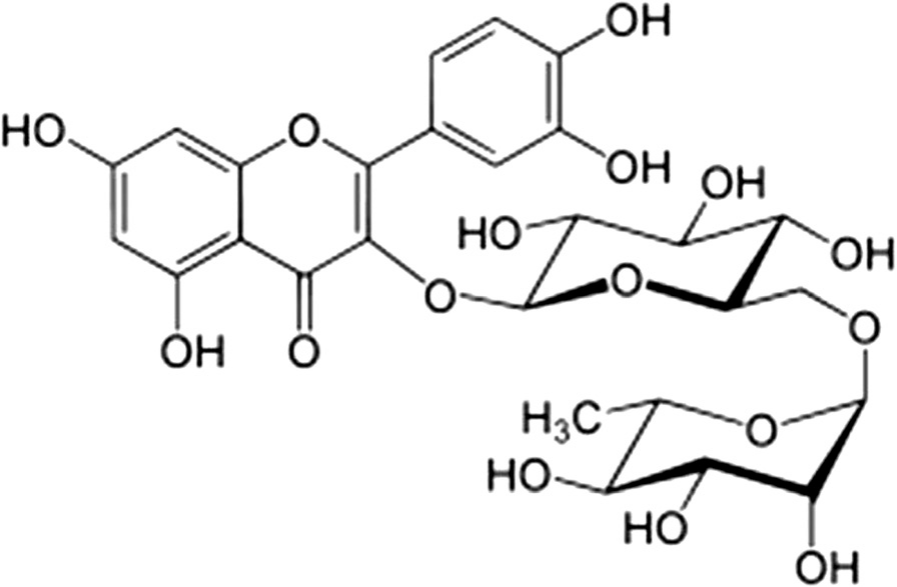
The structure of rutin.

Rutin (Figure 1) works as a scavenger of reactive oxidative species (ROS) by donating hydrogen atoms to peroxy radicals, superoxide anions, and singlet oxygen and hydroxyl radicals; it also functions as a terminator and chelator of metal ions that are capable of oxidizing lipid peroxidation [15-17]. Rutin has been shown to function as an anti-cancer, anti-viral, anti-bacterial and anti-inflammatory agent. It is also used to treat cardiovascular and neurodegenerative disorders because of its appreciable free radical-scavenging and anti-oxidant capacities [18-20]. Additionally, studies suggest that rutin alters signal transduction, causes activation of transcription factors and gene expression, and may also protect DNA by interacting with carcinogens that have escaped detoxification processes [21-23].

The present study was therefore aimed at investigating the effect of rutin on KBrO_3_-induced nephrotoxicity in male Sprague–Dawley rats by the determination of biochemical parameters and by histological examination.

## Methods

### Chemicals

Reduced glutathione (GSH), oxidized glutathione (GSSG), glutathione reductase, gamma-glutamyl p-nitroanilide, glycylglycine, bovine serum albumin (BSA), 1,2-dithio-bis nitro benzoic acid (DTNB), 1-chloro-2,4-dinitrobenzene (CDNB), reduced nicotinamide adenine dinucleotide phosphate (NADPH), CCl_4_, flavine adenine dinucleotide (FAD), glucose-6-phosphate, Tween-20, 2,6-dichlorophenolindophenol, thiobarbituric acid (TBA), picric acid, sodium tungstate, sodium hydroxide, trichloroacetic acid (TCA) and perchloric acid (PCA) were purchased from Sigma Chemicals Co. St. Louis, USA.

### Animals and treatment

Six-week-old male Sprague–Dawley rats weighing 180±10 g were provided with food and water *ad libitum* and kept at 20–22°C on a 12-h light–dark cycle. All experimental procedures involving animals were conducted in accordance with the guidelines of National Institutes of Health (NIH guidelines Islamabad, Pakistan). The study protocol was approved by Ethical Committee of Quaid-i-Azam University, Islamabad. The rats were acclimatized to laboratory condition for 7 days before commencement of experiment.

The following experimental groups (n = 06 rats per group) were studied.

Group I: (control); the animals were remained untreated.

Group II: animals were treated with saline (0.5 ml/kg bw; 0.9% NaCl) intragastric twice a week for four weeks.

Group III: was treated with KBrO_3_ (20 mg/kg bw; aqueous) intragastric twice a week for four weeks.

Group IV: was treated with KBrO_3_ (20 mg/kg bw; aqueous) intragastric and rutin (50 mg/kg bw) intragastric twice a week for four weeks.

Group V: rats were treated with KBrO_3_ (20 mg/kg bw; aqueous) intragastric and rutin (70 mg/kg bw) intragastric twice a week for four weeks.

Group VI: was treated with rutin (70 mg/kg bw) intragastric twice a week for four weeks.

In addition all the rats were provided free access of drinking water and food. After the completion of dosages rats were kept individually in metabolic cages for 24 h; collect the urine and volume was determined. All the animals were sacrificed; blood was drawn prior to the excision of organ. The serum was stored at −80°C after separation until it was assayed as described below. Half of kidney tissues were treated with liquid nitrogen and stored at −80°C for further enzymatic analysis while the other portion was processed for histology.

### Analysis of urine

Urine samples were assayed for pH, specific gravity, urea, creatinine, protein, albumin, urobilinogen, red blood cells (RBCs) and white blood cells (WBCs) count by using standard diagnostic kits (MediScreen Urine Strips, Orgenics, France) and standard AMP diagnostic kits (Stattogger Strasse 31b 8045 Graz, Austria) . Urinary creatinine clearance was estimated by using the formula:

CrCl=UxV/PXT

Where U: concentration of creatinine in urine

P: concentration of creatinine in plasma

V: 24 h of urinary volume

T: Time in minutes

### Analysis of serum

Analysis of serum for blood urea nitrogen (BUN), nitrite, creatinine, total protein, globulin, and albumin was estimated by using standard AMP diagnostic kits (Stattogger Strasse 31b 8045 Graz, Austria).

### Assessment of antioxidant profile

Renal tissue was homogenized in 10 volume of 100 mmol KH_2_PO_4_ buffer containing 1 mmol EDTA (pH 7.4) and centrifuged at 12,000 × g for 30 min at 4°C. The supernatant was collected and used for enzymatic studies. Protein concentration of kidney tissue supernatant was determined by the method of Lowry et al. [24] using crystalline BSA as standard.

### Catalase assay (CAT)

CAT activities were determined by the method of Chance and Maehly [25] with some modifications. The reaction solution of CAT activities contained: 2.5 ml of 50 mmol phosphate buffer (pH 5.0), 0.4 ml of 5.9 mmol H_2_O_2_ and 0.1 ml enzyme extract. Changes in absorbance of the reaction solution at 240 nm were determined after one min. One unit of CAT activity was defined as an absorbance change of 0.01 as units/min.

### Superoxide dismutase assay (SOD)

SOD activity of lung tissues was estimated by the method of Kakkar et al. [26]. Reaction mixture of this method contained: 0.1 ml of phenazine methosulphate (186 μmol), 1.2 ml of sodium pyrophosphate buffer (0.052 mmol; pH 7.0), 0.3 ml of supernatant after centrifugation (1500 × g for 10 min followed by 10000 × g for 15 min) of lung homogenate was added to the reaction mixture. Enzyme reaction was initiated by adding 0.2 ml of NADH (780 μmol) and stopped after 1 min by adding 1 ml of glacial acetic acid. Amount of chromogen formed was measured by recording color intensity at 560 nm. Results are expressed in units/mg protein.

### Estimation of lipid peroxidation assay (TBARS)

The assay for lipid peroxidation was carried out with modified method of Iqbal et al. [27]. The reaction mixture in a total volume of 1.0 ml contained: 0.58 ml phosphate buffer (0.1 mol; pH 7.4), 0.2 ml homogenate sample, 0.2 ml ascorbic acid (100 mmol), and 0.02 ml ferric chloride (100 mmol). The reaction mixture was incubated at 37°C in a shaking water bath for 1 h. The reaction was stopped by addition of 1.0 ml 10% trichloroacetic acid. Following addition of 1.0 ml 0.67% thiobarbituric acid, all the tubes were placed in boiling water bath for 20 min and then shifted to crushed ice-bath before centrifuging at 2500 × g for 10 min. The amount of malonaldehyde formed in each of the samples was assessed by measuring optical density of the supernatant at 535 nm using spectrophotometer against a reagent blank. Tetramethoxypropane was used as an external standard. The results were expressed as nmol of TBARS/min/mg tissue protein.

### Glutathione-S-transferase assay (GST)

The reaction mixture of glutathione-S-transferase activity consisted of 1.475 ml phosphate buffer (0.1 mol, pH 6.5), 0.2 ml reduced glutathione (1 mmol), 0.025 ml (CDNB; 1 mmol) and 0.3 ml of tissue homogenate in a total volume of 2.0 ml. The changes in the absorbance were recorded at 340 nm and enzymes activity was calculated as nmol CDNB conjugate formed/min/mg protein using a molar extinction coefficient of 9.6 × 10^3^M^-1^cm^-1^[28].

### Glutathione reductase assay (GSR)

Glutathione reductase activity was determined with the protocol of Carlberg and Mannervik [29]. The reaction mixture consisted of 1.65 ml phosphate buffer: (0.1 mol; pH 7.6), 0.1 ml EDTA (0.5 mmol), 0.05 ml oxidized glutathione (1 mmol), 0.1 ml NADPH (0.1 mmol) and 0.1 ml of homogenate in a total volume of 2 ml. Enzyme activity was quantitated at 25°C by measuring disappearance of NADPH at 340 nm and was calculated as nmol NADPH oxidized/min/mg protein using molar extinction coefficient of 6.22 × 10^3^ M^-1^cm^-1^.

### Glutathione peroxidase assay (GSH-px)

Glutathione peroxidase activity was assayed by the method of Mohandas et al. [30]. The reaction mixture consisted of 1.49 ml phosphate buffer (0.1 mol; pH 7.4), 0.1 ml EDTA (1 mmol), 0.1 ml sodium azide (1 mmol), 0.05 ml glutathione reductase (1 IU/ml), 0.05 ml GSH (1 mmol), 0.1 ml NADPH (0.2 mmol), 0.01 ml H_2_O_2_ (0.25 mmol) and 0.1 ml of homogenate in a total volume of 2 ml. The disappearance of NADPH at 340 nm was recorded at 25°C. Enzyme activity was calculated as nmol NADPH oxidized/min/mg protein using molar extinction coefficient of 6.22 × 10^3^ M^-1^cm^-1^.

### Reduced glutathione assay (GSH)

1.0 ml sample of homogenate was precipitated with 1.0 ml of (4%) sulfosalicylic acid. The samples were kept at 4°C for 1 h and then centrifuged at 1200 × g for 20 min at 4°C. The total volume of 3.0 ml assay mixture contained: 0.1 ml filtered aliquot, 2.7 ml phosphate buffer (0.1 mol; pH 7.4) and 0.2 ml DTNB (100 mmol). The yellow color developed was read immediately at 412 nm on a SmartSpecTM plus Spectrophotometer. It was expressed as μmol GSH/g tissue [31].

### DNA fragmentation assay

DNA fragmentation assay was conducted using the procedure of Wu et al. [32] with some modifications. The tissue (50 mg) was homogenized in 10 volumes of a TE solution pH 8.0 (5 mmol Tris–HCl, 20 mmol EDTA) and 0.2% triton X-100. 1.0 ml aliquot of each sample was centrifuged at 27,000 × g for 20 min to separate the intact chromatin (pellet, B) from the fragmented DNA (supernatant, T). The pellet and supernatant fractions were assayed for DNA content using a freshly prepared DPA (Diphenylamine) solution for reaction. Optical density was read at 620 nm with (SmartSpecTM Plus Spectrophotometer catalog # 170–2525) spectrophotometer. The results were expressed as amount of % fragmented DNA by the following formula;

$$\%\;Fragmented\;DNA=Tx100/\left(T+B\right)$$

### AgNORs count

Silver staining technique was used according to the Trere et al. [33]. The AgNORs technique was performed on dried slides as follows; unstained fixed slides were dewaxed by dipping for 3 minutes in xylene. After complete removal of wax the slides were hydrated in decrease ethanol concentration (90, 70 and 50%) and washed in distilled water for 10 min and dried in an oven. After drying slides were treated with one drop of colloidal solution (2% gelatin and 1% formic acid) and two drops of 50% AgNO_3_ solution onto the slide and incubated at 35°C for about 8–12 min. The progressive staining was followed under microscope to get golden colored nuclei and brown/black NORs. Then, the slide was washed in distilled water, treated for 1 min with 1% sodium thiosulphate at room temperature to stop the reaction, and washed in tap water. The cells were examined under light microscope at 100 × magnification and number of AgNORs was counted per cell.

### DNA ladder assay

DNA was isolated by using the methods of Gilbert et al. [34]. To estimate DNA damages. 5 μg of rat DNA was separately loaded in 1.5% agarose gel containing 1.0 μg/ml ethidium bromide including DNA standards (0.5 μg per well). Electrophoresis was performed for 45 min at 100 Volt. After electrophoresis gel was studied under gel doc system and was photographed through digital camera.

### RT-PCR (telomerase assay)

Telomerase activity was determined by the protocol of Wen et al. [35] with some modifications.100 mg kidney was washed in ice-cold wash buffer (10 mmol Hepes-KOH pH 7.5, 1.5 mmol MgCl_2_, 10 mM KCl, 1 mM dithiothreitol, 20 μl RNAs inhitors), and homogenised in 200 μl ice cold lysis buffer. The homogenate was incubated on ice for 30 min and then centrifuged at 10,000 ×g for 30 min at 4°C. PCR reaction mixture (total 48 μl) consisted of 36.6 μl DEPC treated water, 2 μl (6 μg protein) extract, 5 μl 10x TRAP reaction solution, 2 μl (50 μmol) each dNTP, 0.4 μl (2 U) Taq DNA polymerase, and 2 μl (0.1 μg) of TS primer sequence (5'-AATCCGTCGAGCAGAGTT-3'). The PCR reaction mixture was incubated at 25°C in water bath for 30 min for extension of TS primer. CX primer sequence (5'-CCCTTACCCTTACCCTTACCCTAA-3') 2 μl (0.1 μg) was added. The reaction mixture (total 50 μl) was subjected to PCR cycles (25) at 94°C for 30s, 55°C for 30s, and 72°C for 90s (then 10 min for the final step). After amplification 5μl of loading dye (0.25% bromophenol blue, 0.25% xylenocyanol and 50% glycerol) was mixed to each PCR product and 25 μl of each sample were loaded onto a 12.5% non-denaturing polyacrylamide gel. After complete running of gel was fixed in fixing solution (0.5% acetic acid, 10% ethanol) and stained with 0.2% AgNO_3_ for 10 min, followed by 15 min incubation in developing solution (0.1% formaldehyde and 3% NaOH) and then photographed.

### Histopathalogical determination

For microscopic evaluation tissues were fixed in a fixative (absolute ethanol 60%, formaldehyde 30%, and glacial acetic acid 10%) and embedded in paraffin, sectioned at 4 μm and subsequently stained with hematoxylin/eosin. Sections were studied under light microscope (DIALUX 20 EB) at 40 and 100 magnifications. Slides of all the treated groups were studied and photographed. A minimum 12 fields of each section were studied and approved by pathologist without saying of its treatment nature.

### Statistical analysis

The values were expressed as the mean ± SEM for the 06 rats in each group. Differences between groups were assessed by one-way analysis of variance (ANOVA) using the Statistical Package for Social Sciences (SPSS) software package for Windows (version 13.0). Post hoc testing was performed for intergroup comparisons using the least significant difference (LSD) test. A value corresponding to P<0.05 was deemed to be statistically significant.

## Results

### Effects of rutin on urine profile

Reactive oxygen species (ROS) especially nephrotoxic chemicals effects the urinary profile of kidney. Table 1 shows the changes in renal profile including pH, specific gravity, RBCs and WBCs count, creatinine, creatinine clearance, urea, urobilinogen, protein, and albumin level. Administration of nephrotoxic KBrO_3_ treatment significantly *(P<0.01)* increased the level of specific gravity, RBCs and WBCs count, creatinine, urea, urobilinogen, protein, and albumin level whereas decreased *(P<0.01)* the pH and creatinine clearance as compared to the control group. Treatment of rutin attenuated the KBrO_3_ intoxication, dose dependently, and decreased *(P<0.01)* the specific gravity, RBCs and WBCs count, urea, creatinine, urobilinogen, protein and albumin while increased the pH and creatinine clearance of urine. Rats treated with rutin (70 mg/kg bw) alone did not statistically (*P>0.05*) change the parameters studied to that of the control group.

**Table 1:** Effects of rutin on physical and biochemical parameters of urine

**Treatment**	**pH**	**Specific gravity**	**Urea (mg/dl)**	**Creatinine (mg/dl)**	**Creatinine clearance (ml/min)**
Control	7.2±0.07^++^	1.2±0.02^++^	93.0±2.0^++^	38.0±1.34^++^	3.4±1.3^++^
Saline (0.5 ml/kg bw; 0.9% NaCl)	7.1±0.04^++^	1.1±0.01^++^	91.0±3.25^++^	36.2±0.09^++^	3.3±0.8^++^
KBrO_3_ (20 mg/kg bw)	6.3±0.08^**^	1.8±0.05 ^**^	195.30±3.98^++^	86.7±3.89^**^	1.1±1.9^**^
KBrO_3_+Rutin (50 mg/kg bw)	6.7±0.10^++^	1.2±0.07^++^	110.0±2.01^*++^	53.5±1.52^++^	2.7±1.6^*++^
KBrO_3_+Rutin (70 mg/kg bw)	7.0±0.03^++^	1.2±0.04^++^	103.1±3.5^++^	46.3±2.31^++^	3.1±1.3^++^
Rutin alone (70 mg/kg bw)	7.1±0.04^++^	1.2±0.06^++^	92.2±3.0^++^	39.1±1.03^++^	3.5±0.3^++^
**Treatment**	**RBC/μl**	**WBC/μl**	**Protein (mg/dl)**	**Albumin (mg/dl)**	**Urobilinogen (mg/dl)**
Control	0.02±0.00^++^	16.0±1.81^++^	42.8±2.3 ^++^	18.2±2.76^++^	2.47±0.22^++^
Saline (0.5 ml/kg bw; 0.9% NaCl)	0.03±0.05^++^	19.6±1.15^++^	43.0±3.2^++^	19.0±3.9^++^	2.43±0.21^++^
KBrO_3_ (20 mg/kg bw)	25.7±1.84^**^	68.0±5.0^**^	100.0±1.2^**^	34.7±1.86^**^	15.7±1.89^**^
KBrO_3_+Rutin (50 mg/kg bw)	8.8±0.15^**++^	41.00±2.25^**++^	55.0±1.2^++^	24.7±1.86^++^	5.67±1.89^++^
KBrO_3_+Rutin (70 mg/kg bw)	2.3±0.12^**++^	22.5±1.3^++^	45.7±2.1^++^	20.6±1.25^++^	3.62±1.23^++^
Rutin alone (70 mg/kg bw)	2.0±0.95^**++^	18.66±0.95^++^	39.0±2.3^++^	17.33±3.71^++^	2.933±0.25^++^

Mean ± SEM (n=6 number).

*, ** indicate significance from the control group at P<0.05 and P<0.01 probability level.

++ indicate significance from the KBrO_3_ group at P<0.01 probability level.

### Effects of rutin on serum profile

Table 2 shows the assessment of serum biomarkers i.e. concentration of nitrite, creatinine, BUN, total protein; albumin and globulin indicate the functional integrity of the kidneys. KBrO_3_ administration significantly *(P<0.01)* increased the level of nitrite, creatinine while significantly *(P<0.01)* decreased the total protein, albumin and globulin as compared to control group. Serum level of these parameters were significantly *(P<0.01)* improved by administration of rutin as compared to KBrO_3_ treated rats. However, more restoration effects on studied parameters were determined at the higher dose (70 mg/kg bw) of rutin. These parameters were statistically (*P>0.05*) remained unchanged with the treatment of rutin (70 mg/kg bw) alone as compared to the control group of rat.

**Table 2:** Effects of rutin on serum profile

**Treatment**	**Nitrite (μmol/ml)**	**Creatinine (mg/dl)**	**BUN (mg/dl)**	**Protein (mg/dl)**	**Globulin (mg/dl)**	**Albumin (mg/dl)**
Control	25.7±1.4^++^	36.55±0.84^++^	41.0±2.04^++^	42.83±2.26 ^++^	24.67±1.33^++^	18.17±2.76^++^
Saline (0.5 ml/kg bw; 0.9% NaCl)	24.9±1.7^++^	34.9±0.24^++^	43.0±2.56^++^	43.6±2.0 ^++^	23.07±1.83^++^	17.10±2.25^++^
KBrO_3_ (20 mg/kg bw)	72.12±3.4^**^	90.0±7.06^**^	125.9±3.5^**^	23.00±1.18^**^	12.33±1.54^**^	10.67±1.86^**^
KBrO_3_+Rutin (50 mg/kg bw)	32.0±2.6^++^	50.3±1.58^++^	65.8±5.58^++^	33.33±1.54^**++^	16.17±1.14^**++^	13.16±1.42^*++^
KBrO_3_+Rutin (70 mg/kg bw)	25.3±2.3^++^	43.6±2.15^++^	52.6±4.62^++^	37.65±2.16^++^	20.42±1.64^++^	16.53±1.26^++^
Rutin alone (70 mg/kg bw)	22.3±1.9^++^	35.17±1.9^++^	40.17±3.75^++^	38.00±2.27^++^	19.67±2.01^++^	17.33±3.71^++^

Mean ±SEM (n=6 number).

** indicate significance from the control group at P<0.01 probability level.

++ indicate significance from the KBrO_3_ group at P<0.01 probability level.

### Effects of rutin on antioxidant profile

The results regarding the protective effects of rutin against the toxic affect of KBrO_3_ in rat on kidney protein and activities of antioxidant enzymes such as CAT, SOD, GSH-Px, GSR and GST are shown in Table 3. Activities of antioxidant enzymes such as CAT, SOD, GSH-Px, GSR and GST were reduced (P<0.01) by treatment of KBrO_3_ as compared to control group. This reduction in enzymes activity was reversed significantly *(P<0.01)*, in a concentration dependent way, by the treatment of rutin as compared to the KBrO_3_ group. Alteration in the renal enzyme activities with the treatment of rutin (70 mg/kg bw) alone was statistically (P>0.05) remained unchanged as compared to the control group of rat.

**Table 3:** Effects of rutin on renal antioxidant profile

**Treatment**	**CAT (U/min)**	**SOD (U/mg protein)**	**GSH-Px (nmol/mg protein)**	**GSR (nmol/min /mg protein)**	**GST (nmol/min/mg protein)**
Control	18.2±2.2^++^	19.05±1.54^++^	45.33±1.65^++^	203.3±10.3 ^++^	123.8±13.3^++^
Saline (0.5 ml/kg bw; 0.9% NaCl)	18.5±2.6^++^	19.75±1.4^++^	43.5±1.35^++^	200.6±8.3 ^++^	121.8±10.3^++^
KBrO_3_ (20 mg/kg bw)	7.5±0.85^**^	7.58±2.08^**^	23.35±3.08 ^**^	114.5±20.7^**^	53.8±26.1^**^
KBrO_3_+Rutin (50 mg/kg bw)	11.5±2.2^*++^	12.83±1.87^*++^	34.91±5.54 ^*++^	158.0±10.5^*++^	104.8±11.5 ^++^
KBrO_3_+Rutin (70 mg/kg bw)	16.5±2.6^++^	17.62±2.14^++^	41.26±4.6^++^	184.5±11.3^++^	116.5±14.3^++^
Rutin alone (70 mg/kg bw)	17.37±1.17^++^	20.72±1.72^++^	46.06±2.12^++^	207.3±13.2^++^	126.2±21.5^++^

Mean ± SEM (n=6 number).

*, ** indicate significance from the control group at P<0.05 and P<0.01 probability level.

++ indicate significance from the KBrO_3_ group P<0.01 probability level.

### Effects of rutin on TBARS, GSH, DNA fragmentation and AgNORs

Table 4 shows the changes of protection by rutin versus the KBrO_3_ intoxication of rat on TBARS, GSH, DNA fragmentation and AgNORs count in kidney of rat. Treatment of KBrO_3_ significantly *(P<0.01)* increased the contents of TBARS, DNA fragmentation and AgNORs count while significantly *(P<0.01)* decreased the content of GSH in kidneys as compared to control group. Concentration of TBARS, AgNORs count, and DNA injuries was significantly decreased *(P<0.01)* by administration of rutin, in a dose dependent way, in rat treated with KBrO_3_. Level of renal GSH was significantly *(P<0.01)* increased by administration of rutin as compared to the KBrO_3_ treated group. However these effects were more pronounced at the higher dose of rutin (70 mg/kg bw). Statistically nonsignificant (P>0.05) alterations on TBARS, GSH, DNA fragmentation and AgNORs count in kidney of rat was determined with rutin (70 mg/kg bw) alone as compared to the control group.

**Table 4:** Effects of rutin on renal level of TBARS, GSH, AgNORs and DNA fragmentation

**Treatment**	**TBARS (nmol/min/mg protein)**	**GSH (μmol/g tissue)**	**DNA fragmentation%**	**AgNORs (NORs/cell)**
Control	8.17±0.93^++^	1.72±0.157^++^	7.75±0.834^++^	1.86±0.10^++^
Saline (0.5 ml/kg bw; 0.9% NaCl)	8.2±0.73^++^	1.68±0.17^++^	7.65±0.94^++^	1.81±0.12^++^
KBrO_3_ (20 mg/kg bw)	20.90±1.91^**^	0.73±0.15^**^	54.42±1.02^**^	8.08±0.52^**^
KBrO_3_+Rutin (50 mg/kg bw)	15.17±1.20^++^	1.28±0.19^*++^	27.85±1.26^++^	2.23±0.09^++^
KBrO_3_+Rutin (70 mg/kg bw)	10.25±1.35^++^	1.58±0.16^++^	9.25±1.35^++^	2.12±0.06^++^
Rutin alone (70 mg/kg bw)	9.17±1.06^++^	1.760±0.0817^++^	8.08±1.17^++^	1.95±0.13^++^

Mean ±SE M (n=6 number).

** indicate significance from the control group at P<0.01 probability level.

++ indicate significance from the KBrO_3_ group at P<0.01 probability level.

### Effects of rutin on percent increase in body weight, absolute kidney weight and relative kidney weight

Protective effects of different dosages of rutin against KBrO_3_ administration to rat on percent increase in body weight, absolute kidney weight and relative kidney weight are shown in Table 5. Administration of KBrO_3_ to rats significantly increased *(P<0.01)* the absolute kidney weight, relative kidney weight while significantly decreased *(P<0.01)* the percent increase in body weight for four experimental weeks as compared to the control group. Administration of rutin consistently restored *(P<0.01)* the percent increase in body weight, kidney weight and relative kidney weight as compared to the KBrO_3_ group. More protective effects of rutin for the percent increase in body weight, kidney weight and relative kidney weight against the KBrO_3_ intoxication to rats were determined at the higher concentration of rutin (70 mg/kg bw). Administration of rutin alone did not cause any significant (P>0.05) change in the percent increase in body weight, absolute kidney weight and relative kidney weight as compared to the control group.

**Table 5:** Effects of rutin on body weight, kidney weight and relative kidney weight of rat

**Treatment**	**Percent increase in body weight (g) after**				**Absolute kidney weight (g)**	**Relative kidney weight (% to bw)**
	**I**^**st**^**week**	**2**^**nd**^**week**	**3**^**rd**^**week**	**4**^**rth**^**week**		
Control	6.3±0.6^++^	13.4±1.3^++^	21.3±2.3^++^	28.2±3.4^++^	4.0±0.08^++^	0.040±0.00072^++^
Saline (0.5 ml/kg bw; 0.9% NaCl)	6.6±0.5^++^	13.8±2.1^++^	22.4±2.6^++^	27.6±3.2^++^	4.0±0.08^++^	0.040±0.00072^++^
KBrO_3_ (20 mg/kg bw)	3.4±0.3^**^	7.6±1.5^**^	10.2±1.6^**^	14.4±2.6^**^	4.9±0.60^**^	0.049±0.00125^**^
KBrO_3_+Rutin (50 mg/kg bw)	4.4±0.3^*++^	10.4±1.2^*++^	15.3±1.4^*++^	19.6±2.4^++^	4.50±0.12^++^	0.045±0.00079^++^
KBrO_3_+Rutin (70 mg/kg bw)	4.9±0.4^++^	12.6±1.4^++^	19.6±1.5^++^	23.4±3.2^++^	4.23±0.14^++^	0.042±0.0014^++^
Rutin alone (70 mg/kg bw)	6.2±0.4^++^	14.8±1.3^++^	22.7±1.6^++^	27.8±2.4^++^	4.12±0.06^++^	0.041±0.00049^++^

Mean ±SEM (n=6 number).

*, ** indicate significance from the control group at P<0.05 and P<0.01 probability level, respectively.

++ indicate significance from the KBrO_3_ group at P<0.01 probability level.

### Effect of rutin on DNA damages (DNA ladder assay)

**Figure 2:**
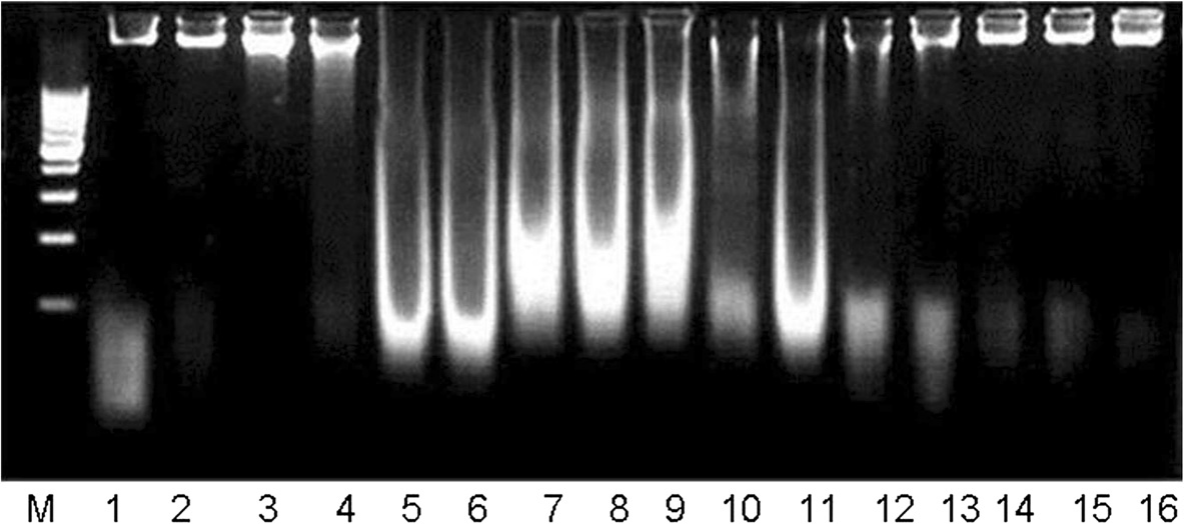
**Agarose gel showing KBrO**_**3**_**-induced DNA damages in kidneys and preventive effects of rutin in various experimental groups of rat.** Lanes (from left) DNA marker (M), Control (1–2), Saline (0.9% NaCl) 0.5 ml/kg bw (3–4), KBrO_3_ 20 mg/kg bw (5–8), KBrO_3_ (20 mg/kg bw) + rutin 50 mg/kg bw (9–11), KBrO_3_ (20 mg/kg bw) + rutin 70 mg/kg bw (12–13), rutin alone 70 mg/kg bw (14–16).

KBrO_3_ forming DNA-free radical adduct, induces DNA damages in the kidneys of rats. DNA ladder assay of kidneys showed the severe DNA damages in the KBrO_3_ treated group of rat. However, administration of rutin to KBrO_3_ treated animals reduced the DNA damages, dose dependently, as shown by DNA banding patterns of different groups of rutin (50 and 70 mg/kg bw) as compared to KBrO_3_ group (Figure 2).

### RT-PCR (telomerase enzyme assay)

**Figure 3:**
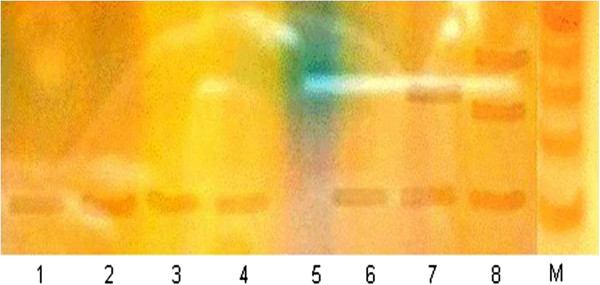
**Polyacrylamide gel shows the telomerase enzyme activity in various groups of the study.** Lane (1–2) control group, Lane (3–4) KBrO_3_ (20 mg/kg bw) + rutin (50 mg/kg bw) group, Lane (5–6) KBrO_3_ (20 mg/kg bw) + rutin (70 mg/kg bw), Lane (7–8) KBrO_3_ (20 mg/kg bw) group.

Telomerase enzyme assay was used to find out the telomerase enzyme activity in various groups of this study (Figure 3). Presence of a single band (primer) in lane (1–2; control group) and in lane (3–6; KBrO_3_ + rutin groups) showed that amplification was absent, and indicated the protective effects of rutin while KBrO_3_ treated group showed positive telomerase activity (lane 7–8) having two or more DNA bands.

### Effects of rutin on histological changes of kidneys

**Figure 4:**
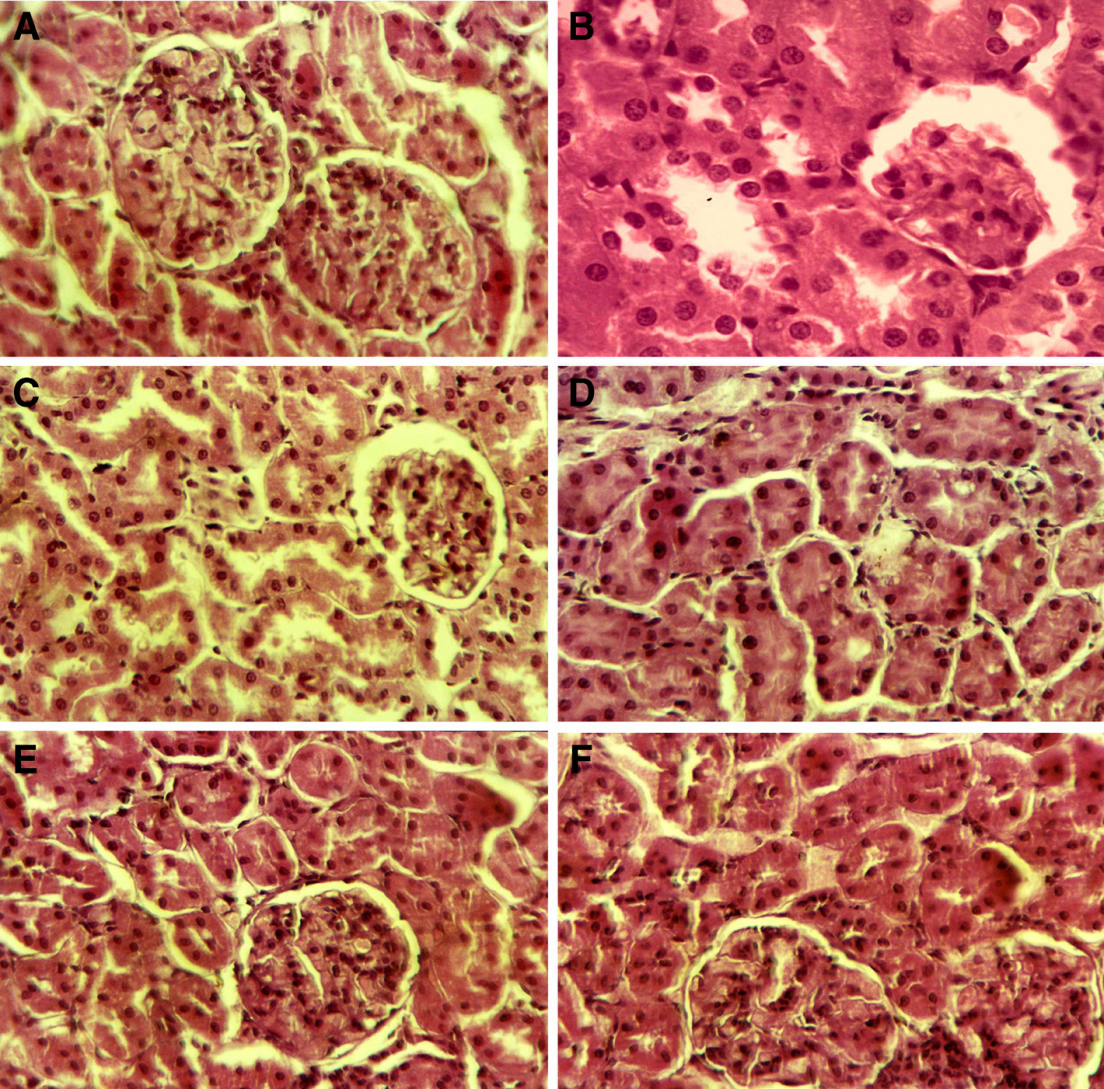
**H & E stain.** Representative section of kidney, Control group; normal tubules, glomerulus and bowman capsule (**A**), KBrO_3_ (20 mg/kg bw) group; injuries in glomeulus and bowman capsule (**B**), KBrO_3_ (20 mg/kg bw) group, tubular necrosis, tubular cell swelling, tubular dilatation (**C**), KBrO_3_ (20 mg/kg bw) group; tubular congestion (**D**), KBrO_3_ (20 mg/kg bw) + rutin (50 mg/kg bw) group (**E**), KBrO_3_ (20 mg/kg bw) + rutin (70 mg/kg bw) group (**F**).

The histological changes were graded and summarized in Table 6. The sections of control group showed normal histology including normal glomerulus, bowman capsule, distal and proximal convoluted tubules. Marked histological changes were observed in cortex of kidneys in KBrO_3_-treated rats. The cross section showed tubular degeneration, tubular congestion, tubular dilatation and glomerular injuries in KBrO_3_-treated rats (Figure 4). Treatment of rutin to KBrO_3_ treated rats markedly recovered the toxic changes near to the control rat. Histology of the kidneys showed normal glomerulus, bowman capsule, reversed the tubular degeneration, congestion and dilatation and prevented interstitial edema and capillary congestion in a dose dependent way. However, treatment of rutin (70 mg/kg bw) alone did not induce histopathological changes in kidneys.

**Table 6:** Effects of rutin on renal histopathology of rat

**Treatment**	**Tubular dilatation**	**Tubular necrosis**	**Tubular cell swelling**	**Tubular congestion**	**Glomerular injuries**
Control	-	-	-	-	-
Saline (0.5 ml/kg bw; 0.9% NaCl)	-	-	-	-	-
KBrO_3_ (20 mg/kg bw)	++	++	++	−/+	−/+
KBrO_3_+Rutin (50 mg/kg bw)	−/+	−/+	−/+	-	-
KBrO_3_+Rutin (70 mg/kg bw)	-	-	-	-	-
Rutin alone (70 mg/kg bw)	-	-	-	-	-

-, normal; −/+, mild; ++, medium damaged.

## Discussion

Rutin modulates several biological functions and exhibits anti-cancer, anti-viral, anti-bacterial and anti-inflammatory activities due to its appreciable free radical-scavenging and anti-oxidant capacities [18-20]. At the end of the study period, KBrO_3_ treatment was found to have decreased the percent increase in body weight but increased the absolute kidney weight and relative kidney weight as compared with the control group. Similar alterations for these parameters with KBrO_3_ have been determined previously [36-38]. In the present study, the observations made with respect to percent increase in body weight, absolute kidney weight and relative kidney weight in male Sprague–Dawley rats appeared to suggest that rutin can alleviate KBrO_3_-induced toxicity in these animals.

Urinalysis provides important clues about acid–base balance and kidney function [39]. Urobilinogen is a conjugated product of bilirubin, which passes through the bile duct and is metabolized in the intestine [40,41]. High levels of urobilinogen, urea, creatinine, protein and albumin in urine reflect the kidney dysfunction and renal injuries induced by KBrO_3_ treatment [42,43]. Increased specific gravity is a basic symptom of dehydration, renal artery steatosis, necrosis, and decreased blood flow to the kidneys. In addition, increased RBC and WBC counts in the urine of KBrO_3_-treated rats suggested severe injuries to renal tissues. Higher levels of protein and the number of RBCs and WBCs might have also contributed to the values of specific gravity obtained in the present study. This increase in specific gravity consequently reflects the high degree of damage to kidney tissue. The hematuria and proteinuria observed in the present study could be related to necrosis and kidney dysfunction [44]. Ogawa et al. [45] also found that the glomerular capillary wall is permeable to low-molecular-weight (LMW) proteins. Therefore, an appreciably high level of proteinuria indicates the leakage of LMW proteins. The oxidative stress induced by KBrO_3_ might promote the formation of various vasoactive mediators that can affect renal function directly by initiating renal vasoconstriction or decreasing the glomerular capillary ultrafiltration coefficient. This action will reduce the glomerular filtration rate, leading to proteinuria. Rutin administration to rats treated with KBrO_3_ ameliorated the toxicity of KBrO_3_ in kidneys to restore the level of the studied parameters in a concentration-dependent manner. These results suggest that rutin can be used as renoprotective agent against KBrO_3_ induced toxicity.

The present study revealed that KBrO_3_ administration caused marked increases in the serum levels of creatinine, urobilinogen, BUN, total bilirubin and direct bilirubin, as reported previously [23,46]. In addition, elevated levels of urinary albumin and reduced levels of serum albumin in KBrO_3_-treated rats might have resulted from considerable leakage due to injuries to glomeruli and tubules. The results suggested that rutin prevented KBrO_3_-induced toxicity, and that the levels of creatinine, urobilinogen, BUN, total bilirubin, and direct bilirubin could be altered to those seen in the control group. Other studies have shown that different plant extracts can significantly reduce the renal injuries induced through KBrO_3_ intoxication [12,23,43]. There is a large body of evidence implicating oxidative stress and ROS in the mechanism of KBrO_3_-induced toxicity in animal models [10]. In the present study, the mean activity of the antioxidant enzymes CAT, SOD, GSH-Px, GST and GSR were found to be significantly lowered in the KBrO_3_-treated group compared with that of the control group. Lowered activities of these antioxidant enzymes with KBrO_3_ in *in-vivo* experimental models have been reported [47]. However, the treatment of rutin with KbrO_3_ modified the biochemical changes caused by KBrO_3_ in rat. In the present study, the mean activities of antioxidant enzymes were significantly higher compared with those of the KBrO_3_-treated group and thus had a potential protective effect.

GSH is a vital extracellular and intracellular protective antioxidant against oxidative stress. It reduces hydrogen peroxides and hydroperoxides by its redox and detoxification reactions, and protects protein thiol groups from oxidation. This tripeptide is present in high concentrations in kidney cells. In the present study, the mean level of GSH upon KBrO_3_ treatment was depleted in the kidneys compared with that seen in the control group. Following decreases in the level of GSH, oxidative stress increases and, thereafter, cell damage occurs [47]. In the present study, the animals receiving KBrO_3_ showed glomerular injuries, tubular necrosis, tubular dilatation, tubular cell swelling and tubular brush border loss. Endogenous levels of GSH were found to be increased with an accompanying increase in the mean activities of GSH-Px, GST and GSR with rutin to that of the KBrO_3_-treated group. By the catalyses of GSH-Px, GSH is oxidized to GSSG, which can then be reduced back to GSH by GSR. GSH is also a cofactor for the phase-II enzyme GST, which confers protection against toxic chemicals by catalyzing the formation of GSH-electrophile conjugates [48].

Free radicals and reactive oxygen species mediate the propagation of peroxidation of polyunsaturated fatty acids, this cascade can be prevented through enzymatic and non-enzymatic antioxidants. Increased TBARS concentration of renal tissues in KBrO_3_ treated rat may be result of increased oxidative stress. TBARS, the final metabolite of peroxidized polyunsatured fatty acids [49], considered as a late biomarker of oxidative stress [50-52], not only translate reactive oxygen species into active chemicals but also magnifies the function of reactive oxygen species through the chain reaction, inducing alterations in cellular and functional impairment [53], and serves to indicate the presence of free radicals, lipid peroxide formation [54,55]. The increment in lipid peroxidation as assessed by the elevated levels of TBARS following KBrO_3_ administration has been well documented in other studies [39,56]. This may be the consequence of an increment in the formation of oxygen free radicals (generated by KBrO_3_) since antioxidant defense systems are compromised [10]. Lower concentration of TBARS with rutin in KBrO_3_ treated animals obtained in this study indicates the ameliorating effects of rutin against the oxidative stress induced with KBrO_3_ in kidneys.

Oxidative damage to DNA is a result of interaction of DNA with reactive oxygen species (ROS), in particular the hydroxyl radical, produces a multiplicity of modifications in DNA; generating strand breaks with various sugar modifications, and release of free bases from nucleic acid. The present study showed increased percentage of DNA fragmentation in the KBrO_3_ treated rat kidneys. This reveals that KBrO_3_ induces oxidative stress to the cells thus causing damage to DNA. Increase in oxidative status with KBrO_3_ in kidneys can permit further oxidation of cellular DNA resulting in the formation of the mutagenic lesion, 8-oxodeoxyguanosine [6,49]. GSH and other sulfhydryls play a major role in the excretion of bromate in the rat kidney [3]. Thiol-mediated oxidation of DNA by bromine oxides and bromine radicals has been well characterized *in vitro* and are thought to play a role in DNA damage *in vivo*[5], while extra cellular pools of GSH may be important in protecting target organs from bromate uptake and oxidative DNA damage [39], intracellular GSH may facilitate the formation of DNA reactive metabolites [57].

Telomeres are specialized structures present at the ends of chromosomes possessing highly conserved G rich sequence TTAGGG in all eukaryotic cells that are gradually decreases during cell division of cell cycle. Telomerase is a key enzyme for synthesis of telomeric repeats and is used as a diagnostic tool for malignant tumors [58,59] and in cell lines [60]. Activity of telomerase was found positive in KBrO_3_ treated kidneys indicating the involvement of oxidative stress in the activation of telomerase. However, telomerase activity was not determined in control as well as in rutin treated groups, which might be due to the presence of various telomerase and cancer inhibitor compounds [61,62]. Similar results have been documented in different studies [63,64]. Renal dysfunction induced by KBrO_3_ in experimental animals characterized by tubular damage, loss of brush border, tubular necrosis, tubular dilatation, tubular cell swelling and glomerular injuries [44,65]. It is believed that these histopathological changes can alter the capacity of tubular absorption, thus bringing about functional overload of nephrons with subsequent renal dysfunction [12,23], which was normalized by co-treatment with rutin. Similar observations were also observed by co-treatment with caffeic acid phenyl ester [43].

## Conclusion

The protective role of rutin at different levels was evaluated in this manuscript. It may contribute its protective effects by erasing the damaging action of potassium bromate at various metabolic cycles, and the repair of DNA damage. The protective potential may involve scavenging potential and antioxidant capacity to ameliorate the KBrO_3_ induced toxicity. This study substantiated the scientific evidence in favors of its pharmacological use in renal injuries.

## Competing interests

The authors declare that they have no competing interests.

## Authors’ contributions

RAK made a significant contribution to acquisition of data, analysis, and revising the manuscript for intellectual content. MRK and SS help in data analysis and drafting. The authors read and approved the final manuscript.

## Pre-publication history

The pre-publication history for this paper can be accessed here:

http://www.biomedcentral.com/1472-6882/12/204/prepub
